# Supplementary material for: Influence of Heat Accumulation on Morphology Debris Deposition and Wetting of LIPSS on Steel upon High Repetition Rate Femtosecond Pulses Irradiation
Source: Materials (Basel). 2022 Oct 25;15(21):7468. doi: 10.3390/ma15217468 (PMC9656394; doi:10.3390/ma15217468)
Supplement: Supplementary file 1 [file materials-15-07468-s001.zip › materials-1939121-supplementary.pdf]

Influence of heat accumulation on morphology debris deposition and wetting of LIPSS on steel upon high repetition rate femtosecond pulses irradiation

Camilo Florian[1,*], Yasser Fuentes-Edfuf[1], Evangelos Skoulas[2], Emmanuel Stratakis[3], Santiago Sánchez-Cortés[3], Javier Solis[1], Jan Siegel[1,*]
1. Instituto de Óptica (IO-CSIC), Consejo Superior de Investigaciones Científicas (CSIC), Serrano 121, 28006 Madrid, Spain
2. Instituto de Estructura de la Materia (CSIC), Consejo Superior de Investigaciones Científicas, Serrano 121, 28006, Madrid, Spain
3. Institute of Electronic Structure and Laser (IESL), Foundation for Research and Technology (FORTH), N. Plastira 100, Vassilika Vouton, 70013, Heraklion, Crete, Greece
*Corresponding authors: camilo.florian@csic.es

Supplementary Material

Forward differencing in Time and Central differencing in Space" FDTC method

The method used here considers a two-dimensional adaptation of the finite-difference method for linear boundary-value problems, that requires a 2-dimensional grid defined in a horizontal interval [*a,b*] and a vertical interval [*c,d*]. These intervals are partitioned vertically into *n*-equal parts and horizontally by *m*-equal parts vertically. This grid is already shown in Figure 8, where the vertical axis refers to the time evolution in steps $dt = 1$ ns, and the horizontal axis refers to positions along a 1-dimensional bar made of steel, divided into cells of size $dx = 15$ nm.

Assuming that the material is isotropic, the thermal conductivity at each point in the body is independent of the direction of the heat flow through the point. Suppose that k, c and ρ are functions of (x, y, z) for the 3-dimensional problem, representing the thermal conductivity, specific heat and density respectively at a point (x, y, z). The temperature $u = u(x, y, z, t)$ in a body can be found by solving the partial differential equation:

$$\frac{\partial}{\partial x}\left(k\frac{\partial u}{\partial x}\right)+\frac{\partial}{\partial y}\left(k\frac{\partial u}{\partial y}\right)+\frac{\partial}{\partial z}\left(k\frac{\partial u}{\partial z}\right)=c\rho\frac{\partial u}{\partial t} \qquad \text{(S1)}$$

When k, c and ρ are constants, this equation is known as the simple 3-dimensional heat equation expressed as:

$$\frac{\partial^2 u}{\partial x^2}+\frac{\partial^2 u}{\partial y^2}+\frac{\partial^2 u}{\partial z^2}=\frac{c\rho}{k}\frac{\partial u}{\partial t} \qquad \text{(S2)}$$

Since in most situations k, c and ρ are not constant, and the boundaries are irregular, the solution to this partial differential equation must be obtained by approximation techniques. We assume that since the material is isotropic, the solution for a 1-dimensional bar of certain length l = 600 μm could be extrapolated under some specific conditions; the rod has a uniform temperature within each cross-sectional element dx, the rod is perfectly insulated on its lateral surface, and certain constant α is determined by the heat-conductive properties of the material of which the rod is composed, assumed to be independent of the position in the rod. The parabolic partial differential equation we consider is the following:

$$\alpha^2 \frac{\partial^2 u}{\partial x^2}(x,t) = \frac{\partial u}{\partial t}(x,t) \qquad \text{(S3)}$$

One of the typical sets of constraints for a heat-flow problem of this type is to specify the initial heat distribution in the rod, as $u(x, 0) = f(x)$, and to describe the behaviour at the ends of the rod, known as boundary conditions. In our specific case, the ends of the rod are insulated and are held at constant temperature $U_1 = U_2 = 273$ K, therefore $u(0, t) = u(l, t)$, and correspondingly:

$$\frac{\partial u}{\partial x}(0,t) = 0 \; and \; \frac{\partial u}{\partial x}(l,t) = 0 \qquad \text{(S4)}$$

Then, no heat escapes from the rod and in the limiting case the temperature on the rod is constant. Every time a pulse arrives at the rod, the heat distribution function f(x) corresponds to a Gaussian distribution with dimensions and amplitude equal to the ones used during the experiments as indicated in Materials and Methods section.

The approach to approximate the solution to this simplified problem involves finite differences. We use as integer an $m > 0$, in our specific case m = 40,000 and the x-axis step size dx = l/m = 15 nm. The grid points in this situation are (x_i, t_j), where $x_i = idx$, for i = 0, 1, …, m, and $t_j = jdt$, for j = 0, 1, …n whose maxima are different for each case depending on the repetition rate and scan speed used for computing optimization. We implement the forward difference method using the Taylor series in t, to form the difference quotient:

$$\frac{\partial u}{\partial t}(x_i, t_j) = \frac{u(x_i,t_j+dt)-u(x_i,t_j)}{k} - \frac{k}{2}\frac{\partial^2 u}{\partial t^2}(x_i, \mu_j) \quad \text{(S5)}$$

For some $\mu_j \in (t_j, t_{j+1})$, and the Taylor series in x to form the difference quotient

$$\frac{\partial^2 u}{\partial x^2}(x_i, t_j) = \frac{u(x_i+dx,t_j)-2u(x_i,t_j)+u(x_i-dx,t_j)}{dt^2} - \frac{dt^2}{2}\frac{\partial^4 u}{\partial x^4}(\xi_i, t_j) \quad \text{(S6)}$$

where $\xi_i \in (x_{i-1}, x_{i+1})$. This parabolic partial differential equation implies that at interior gridpoints (x_i, t_j), for each i = 1,2,…,m and j = 1,2,… n, we have

$$\frac{\partial u}{\partial t}(x_i, t_j) - \alpha^2 \frac{\partial^2 u}{\partial x^2}(x_i, t_j) = 0 \quad \text{(S7)}$$

Therefore, the forward difference quotients of equations (5) and (6) are:

$$\frac{w_{i,j+1}-w_{ij}}{dt} - \alpha^2 \frac{w_{i+1,j}-2w_{ij}+w_{i-1,j}}{dx^2} = 0 \quad \text{(S8)}$$

Where w_{ij} approximates $u(x_i, t_j)$.

The explicit nature of this difference method implies that the $(m\text{-}1)\times(m\text{-}1)$ matrix associated with this system can be written in the tridiagonal form:

$$A = \begin{bmatrix} (1-2\lambda) & \lambda & 0 & \dots & 0 \\ \lambda & (1-2\lambda) & \lambda & \ddots & \vdots \\ 0 & \ddots & (1-2\lambda) & \ddots & 0 \\ \vdots & \ddots & \ddots & \ddots & \lambda \\ 0 & \dots & 0 & \lambda & (1-2\lambda) \end{bmatrix} \quad \text{(S9)}$$

Where $\lambda = \alpha^2 (dt/dx^2)$. If we let:

$$\mathrm{w}^{(0)} = (w_{1j,} w_{2j,} \dots, w_{m-1,j,})^t \quad \text{(S10)}$$

for each $j = 1, 2, \ldots$, then the approximate solution is given by:

$$\mathrm{w}^{(j)} = A\mathrm{w}^{(j-1)} \tag{S11}$$

for each $j = 1, 2, \ldots n$, so $\mathrm{w}^{(j)}$ is obtained from $\mathrm{w}^{(j-1)}$ by a simple matrix multiplication. This is known as the Forward-Difference method, and the approximation shown in Figure 8 uses information from the other previous points marked on that figure, as indicated in the text.

Reference

[1] Burden, R. L., Faires, D. Chapter 12: Numerical solutions to partial differential equations, in *Numerical Analysis,* 9th ed.; Julet, M., Brooks/Cole Cengage Learning, Boston, MA, USA, 2010; pp 713-773.
